# Genome Sequence of CaiB, a DR Cluster Actinobacteriophage That Infects Gordonia rubripertincta

**DOI:** 10.1128/mra.00376-22

**Published:** 2022-06-27

**Authors:** Bienna Welsh, Nader M. Abdalla, Esteban Aldana, Veronica M. Alvarado Fernandez, Bruna Arenales Salgado de Oliveira, Diane Fakhre, Amelia J. Haymond, Katelyn M. Helton, Aditi Kanchibhatta, Jahwanza Knight, Sydney Marshall, Maomi Laine N. Martinez, Arielle Merkher, Savannah E. Morrow, Katie P. Nguyen, Jahanvi J. Patel, Somesh R. Patel, Pravalika Rayala, Kira M. Ruiz-Houston, Aarya P. Satardekar, Shifa M. Shaikh, Adrian E. Terron Osorio, Rachel C. Weitz, Louis Otero, Richard S. Pollenz

**Affiliations:** a Department of Cell Biology, Microbiology, and Molecular Biology, University of South Florida, Tampa, Florida, USA; Queens College CUNY

## Abstract

CaiB is a DR cluster actinobacteriophage that was isolated from soil in Florida using Gordonia rubripertincta NRRL B-16540 as the host. The genome is 61,620 bp, has a GC content of 68.6%, and contains 85 predicted protein coding genes. CaiB has several putative operons and has repeated intergenic regions that may be involved in gene regulation.

## ANNOUNCEMENT

There are an estimated 10^31^ phage particles in the world, and 4,000 actinobacteriophage genomes have been annotated (1). Since approximately 70% of annotated protein coding genes do not have known function (2), the isolation of evolutionarily diverse actinobacteriophages helps advance the understanding of phage genomics and evolutionary science.

CaiB was isolated from a moist soil sample from Tampa, Florida (28.086388N, 82.384166W), using Gordonia rubripertincta NRRL B-16540 as the host. Bacterial infections were performed at 30°C utilizing peptone-yeast calcium agar (PYCa). Genomic DNA was isolated after three rounds of plaque purification using the Wizard DNA cleanup kit (A7280; Promega). Genomic DNA was used to create sequencing libraries with NEBNext Ultra II library preparation kit v3 reagents. Sequencing was performed by the Pittsburgh Bacteriophage Institute, and the libraries were run on an Illumina MiSeq instrument, yielding 295,653 paired-end 150-base reads with 625-fold average coverage. Raw reads were assembled with Newbler v2.9 (3), yielding a single phage contig. The results were checked for completeness, accuracy, and genome termini using Consed (4). Default parameters were used for all software unless otherwise specified. CaiB is circularly permuted based on a lack of defined genome ends (5) and was bioinformatically linearized such that base 1 is assigned in accord with other *Gordonia* phages (5). CaiB was autoannotated using DNA Master v5.23.6 (6), and all of the genes were then manually validated for correct starts and functional calls. GeneMark v2.5 (7) and Glimmer v3.02 (8) were utilized to assess start sites and coding potential, and Starterator v1.2 (2) was used to summarize the starts across each family of phage genes. To collect evidence for gene function and the validity of each gene product, HHpred v3.2 (9), NCBI BLAST (10), the Conserved Domain Database (CDD) (11), TMHMM v2.0 (12), and SOSUI (13) were utilized. tRNAscan-SE v2.0 (14) and ARAGORN v1.2.41 (15) were utilized to identify putative tRNAs and transfer-messenger RNAs.

**FIG 1:**
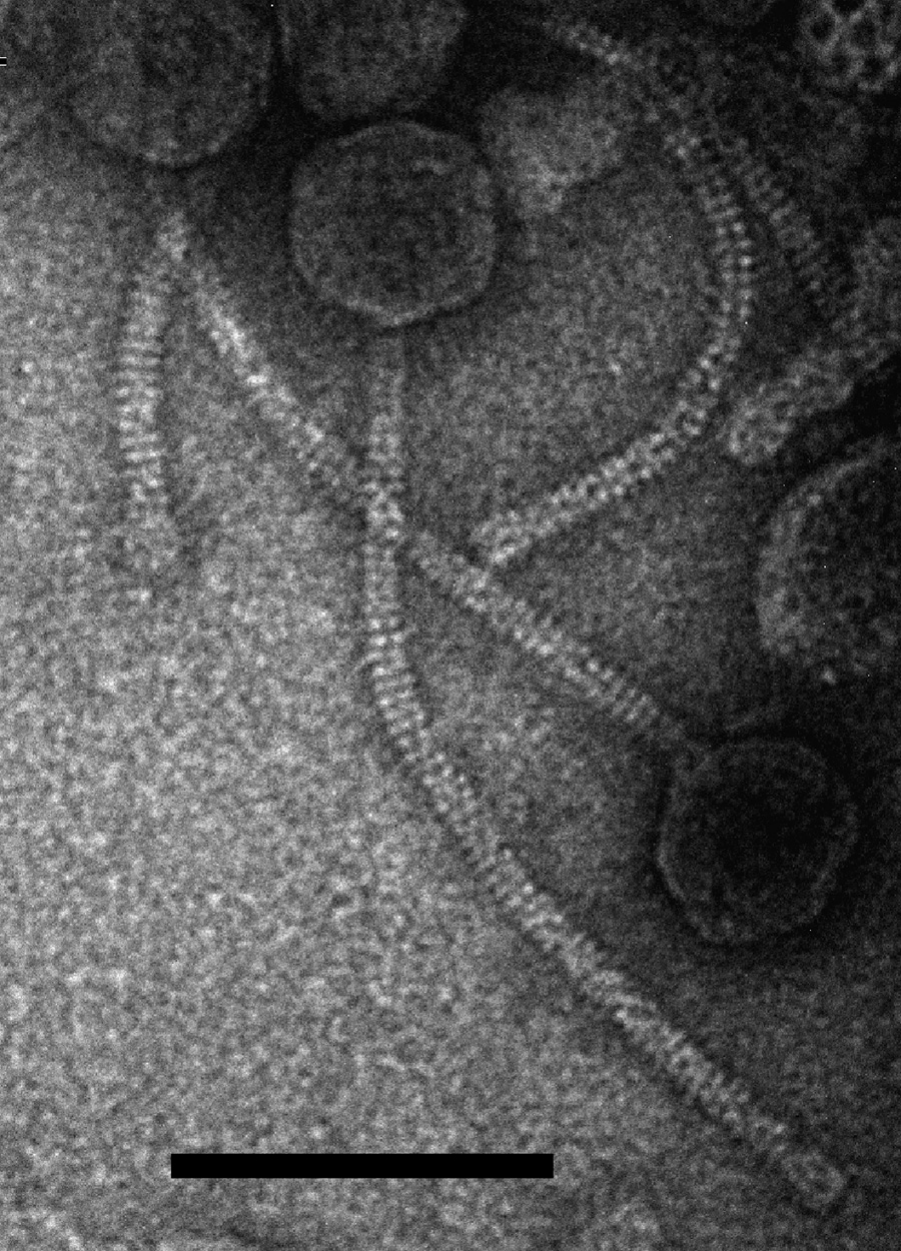
Transmission electron micrograph of *Gordonia* phage CaiB (https://phagesdb.org/phages/CaiB). Phage lysates were negatively stained with 1% uranyl acetate. Scale bar = 120 nm.

Negative-staining transmission electron microscopy shows that CaiB has a 310-nm tail and icosahedral capsid of 62 nm (Fig. 1). CaiB has a 61,620-bp genome, has a GC content of 68.6%, and contains 85 predicted protein coding genes. CaiB is one of 11 phages in the DR cluster (Table 1). DR phages have similar genome organizations and morphologies. The CaiB genome shows between 65 and 97% nucleotide identity to the other DR members (Table 1) (16). Genes 1 to 5 precede the terminase, have 4-bp overlaps, and encode a series of nucleotide modification enzymes, including ParB-like nuclease, TET/JBP oxidoreductase, pyrophosphorylase, and adenylate kinase, which may be involved in evasion of host restriction systems (17). CaiB contains several repeated intergenic regions upstream of genes 42, 71, 77, 80, 81, and 85. These regions contain 100% identity in positions −4 to −11 (5′-GAGAGGAC-3′), −14 to −21 (5′-ACCCGCTC-3′), −25 to −30 (5′-GCGGGA-3′), and −35 to −40 (5′-TGTTGT-3′) and may serve a function in regulation.

**TABLE 1:** DR cluster phage characteristics and nucleotide identity values

Phage	GenBank accession no.	Genome size (bp)	GC content (%)	Nucleotide identity (%) with:										
				NHagos	CaiB	AnarQue	CloverMinnie	Mariokart	Axumite	Ligma	Sour	BiggityBass	AnClar	Yago84
NHagos	MN369758	59,580	68.2	100										
CaiB	ON108644	61,620	68.6	75.88	100									
AnarQue	OK216879	61,822	68.8	76.19	96.96	100								
CloverMinnie	MN234196	61,098	68.7	76.23	96.94	97.98	100							
Mariokart	MT657335	60,762	70.5	67.29	66.44	66.72	66.81	100						
Axumite	ON081333	61,714	70.2	67.44	65.99	66.31	66.43	81.79	100					
Ligma	OM105886	61,714	70.2	67.44	65.99	66.31	66.43	81.79	99.99	100				
Sour	MH153810	61,670	68.0	64.11	64.70	64.86	64.97	71.01	71.62	71.62	100			
BiggityBass	ON260813	63,202	69.4	66.16	65.23	65.30	65.43	73.47	74.76	74.76	72.74	100		
AnClar	MN908693	61,856	69.8	66.03	65.14	65.22	65.29	73.31	74.75	74.76	73.04	88.56	100	
Yago84	MK801725	61,890	70.0	66.00	65.18	65.30	65.37	73.06	74.55	74.55	73.97	89.30	98.49	100

### Data availability.

This whole-genome shotgun project has been deposited in DDB/ENA/GenBank under the accession numbers ON108644 and SRX14597705. The version described in this paper is the first version. Data for CaiB are archived in Phamerator (18) and the Actinobacteriophage Database at PhagesDB.org (2) (https://phagesdb.org/phages/CaiB).
